# Manifestations of Indirect Self-Destructiveness and Dimensions of Emotional Intelligence

**DOI:** 10.1007/s11126-015-9396-9

**Published:** 2015-10-09

**Authors:** Konstantinos Tsirigotis, Joanna Łuczak

**Affiliations:** Department of Psychology, The Jan Kochanowski University in Kielce, Piotrków Trybunalski Branch, Słowackiego 114/118 Str., 97-300 Piotrków Trybunalski, Poland

**Keywords:** Manifestations of indirect self-destructiveness, Dimensions of emotional intelligence, Relationships

## Abstract

While indirect self-destructiveness exerts a rather negative influence on the life and psychological and social functioning of the individual, emotional intelligence may have a favourable effect. The aim of this study has been to explore possible relationships between manifestations of indirect self-destructiveness and dimensions of emotional intelligence. A population of 260 individuals (130 females and 130 males) aged 20–30 (mean age of 24.5) was studied by using the Polish version of the Chronic Self-Destructiveness Scale and INTE, i.e., the Polish version of the Assessing Emotions Scale. Manifestations of indirect self-destructiveness show many significant correlations with variables of the INTE, and those correlations are negative. Generally, it can be said that low emotional intelligence is associated with poor psychosocial and social functioning, which, in turn, is associated with indirect self-destructiveness and its manifestations. It seems advisable to use emotional intelligence in the prophylactic and therapeutic work with individuals suffering from various types of disorders, especially the syndrome of indirect self-destructiveness.

## Introduction

It is a well-known fact that not all behaviours displayed by the human bring about positive and beneficial effects for him or her. Many behaviours may lead to harmful, adverse consequences for the human’s present or subsequent physical, mental and social condition. In psychology, such behaviours are called self-destructive behaviours. Two basic forms of self-destructive behaviours can be distinguished: direct (open, acute) and indirect (latent, chronic) [1–3].

A majority of authors usually consider “self-destructive behaviours” to be behaviours categorised as directly self-destructive, most commonly self-mutilation, self-inflicted injury, and attempted or committed suicide. Literature usually offers studies into direct self-destructiveness (self-mutilation, self-inflicted injury, attempted suicide, committed suicide) or into specific and separate behaviours being manifestations of what is nowadays called indirect or chronic self-destructiveness.

Kelley describes chronic self-destructiveness as a generalised tendency to undertake behaviours increasing the probability of negative and decreasing the probability of positive consequences for the subject [1]. For the purposes of this study, it was assumed that indirect/chronic self-destructiveness comprises behaviours whose probable negative effect is intermediated by additional factors, while the relationship between a behaviour and harm is perceived as probable. Indirect self-destructiveness understood in such a way includes both taking and abandoning specific actions; it concerns getting into hazardous and increased-risk situations (active form) or neglecting one’s safety or health (passive form). Moreover, indirect self-destructiveness is a form of self-destruction characterised by an increased temporal distance between an action and its effect [2, 3]. There are, in general, several categories of indirectly self-destructive behaviours: transgression and risk, poor health maintenance, personal and social neglects, lack of planfulness, and helplessness and passiveness when facing problems/difficulties. Transgression and risk are behaviours violating social norms, such as school rules or principles of community life, as well as risky behaviours undertaken for a momentary pleasure, e.g., driving with bravado connected with a desire to impress others, feel appreciated, better or noticed, or gambling. That category also comprises succumbing to temptations, impulsiveness, and seeking risky excitation. Poor health maintenance encompasses behaviours harmful to one’s health, such as excessive eating or drinking, missing medical appointments or ignoring physicians’ instructions. Personal and social neglects include, for instance, neglecting one’s duties or matters (personally and interpersonally) important to the subject. Lack of planfulness consists in acting mainly on the spur of the moment with nothing in view. Helplessness and passiveness mean giving up an action or not taking that in circumstances where such an action might stop suffering or prevent a danger [1–3].

Indirect self-destructiveness is a form of harming oneself that distinctly differs from direct self-destructiveness or self-aggression. The essence of indirect self-destructiveness is its trans-situational nature and the co-occurrence of various forms of behaviours that lead to adverse consequences. It is not a coincidence that indirect-self destructiveness is referred to as “slow” or “lingering” suicide.

The authors of the article wish to identify selected factors creating favourable conditions for humans to exhibit self-destructive behaviours in order to, through their identification, plan prophylactic and therapeutic measures. Among numerous psychological constructs defined on the basis of research as beneficial and favourable, a lot of weight is attached to emotional intelligence as a trait having adaptive significance and constituting a specific resource of the individual. The second half of the 20th century saw the occurrence of hypotheses proposing that emotions may positively affect mental processes and psychological functioning in general [cf. 4].

According to Salovay and Mayer’s model, emotional intelligence is a set of abilities and a subset of social intelligence that includes the following three categories of adaptive abilities: appraisal and expression of emotions, regulation of emotions, and utilisation of emotions in problem solving. The first category consists of components of appraisal and expression of one’s own emotions and appraisal of emotions of others. The component of appraisal and expression of one’s own emotions is further divided into two subcomponents, i.e.: verbal and non-verbal, while the component of appraisal of emotions of others is divided into subcomponents of non-verbal perception and empathy. The second category of emotional intelligence—regulation—includes components of regulation of emotions in self and regulation of emotions in others. The third category—utilisation of emotions—incorporates components of flexible planning, creative thinking, redirected attention and motivation. Even though emotions are at the core of the model, it also includes social and cognitive functions connected with expression, regulation and utilisation of emotions [4, 5]. Mayer et al. [6] further developed that model but, in the opinion of many authors, fundamental aspects of emotional intelligence proposed in the latest model are similar to those contained in the 1990 one [cf. 7].

Consequently, individuals who have developed abilities connected with emotional intelligence understand and express their own emotions, recognise emotions of others, regulate affect and utilise moods and emotions to motivate adaptive behaviours [4]. Authors wonder whether it is not yet another definition of a healthy, self-actualising individual.

Research has shown that individuals who are primarily motivated by current emotional factors are more likely than those motivated by more distant cognitive considerations to engage in acts that are ultimately self-destructive. Generally, those individuals who are more responsive to immediate emotional factors than to more distant rational projections of consequences are likely to engage in maladaptive acts. Though the specific acts in question vary widely, the unifying characteristic seems to be response to affect rather than to cognitions. Every behaviour appears to represent the tendency to seek immediate pleasure or avoid immediate discomfort, regardless of the long-term consequences of doing so [1].

While emotional intelligence may have a favourable influence on the life and psychological and social functioning of the individual, indirect self-destructiveness exerts a rather negative influence. World literature offers almost no studies into relationships between indirect self-destructiveness and emotional intelligence. As a result of recently carried out research, it was found that indirect self-destructiveness as a generalised behavioural tendency negatively correlates with emotional intelligence [8].

The aim of this study has been to explore possible relationships between individual manifestations of indirect self-destructiveness and particular dimensions of emotional intelligence.

## Methods

The study is part of two more extensive research projects (on indirect self-destructiveness and on emotional intelligence) and thus the applied methodology or some other parts may be similar.

## Participants

A population of 260 individuals (130 females and 130 males) aged 20–30 (mean age of 24.5) was studied by using the Polish version of the Chronic Self-Destructiveness Scale (CS-DS) by Kelley et al. [1] in its adaptation by Suchańska [2] and the Polish version of the Assessing Emotions Scale (AES) by Schutte et al. [5] in its adaptation by Ciechanowicz, Jaworowska and Matczak [9]. The study group was formed on the basis of random selection from the general population (of healthy subjects); participation in the study was voluntary and anonymous, and consistent with the principles of the Declaration of Helsinki.

## Materials

In order to examine indirect (chronic) self-destructiveness as a generalised tendency, Kelley created a research tool comprising several categories of indirectly self-destructive behaviour; the ultimate version was made up of a Likert-type internally consistent set of 52 items with the total obtained score indicating the intensity of indirect self-destructiveness. The research tool encompasses the following categories: Transgression and Risk (A1), Poor Health Maintenance (A2), Personal and Social Neglects (A3), Lack of Planfulness (A4), and Helplessness and Passiveness in the face of problems/difficulties (A5), the scores for which sum up to one global score for indirect self-destructiveness. Both the original scale and its Polish adaptation are characterised by high reliability and validity [1, 2].

Schutte et al. [5] created a tool to examine emotional intelligence. Since then, the questionnaire has been used in many studies, although under different names [7, 10–13]. This study applies the Emotional Intelligence Questionnaire INTE, i.e., the Polish version of AES, as adapted by Ciechanowicz, Jaworowska and Matczak [9]. The questionnaire is composed of 33 items on which the subject may take a position by choosing one of the five possible answers (the Likert-type scale). Along with the general emotional intelligence score, the scale enables to receive scores for two factors: Factor I is ability to utilise emotions in order to support thinking and actions, while Factor II is ability to recognise emotions. Both the original and Polish versions are characterised by high reliability and validity [5, 9].

## Statistical Analysis

The statistical analysis of received scores applied descriptive methods and statistical inference methods. In order to describe the mean value for quantitative traits, the arithmetic mean (M) was calculated, while the standard deviation (SD) was assumed to be the dispersion measure. The conformity of distributions of quantitative traits with the normal distribution was assessed using the Shapiro–Wilk test. Due to the lack of conformity of distributions of dependent variables with the normal distribution, the statistical processing of received results used non-parametric statistics; in order to examine the relationship between the studied variables Kendall’s “tau” (*τ*) correlation coefficient was used. For all the analyses, the maximum acceptable type I error was assumed at α = 0.05. Asymptotic two-sided probability test p was calculated and p ≤ 0.05 was considered to indicate statistical significance. The statistical analyses were performed by means of the *Statistica PL 10.0 for Windows* [14] statistical package.

## Results

**Fig. 1:**
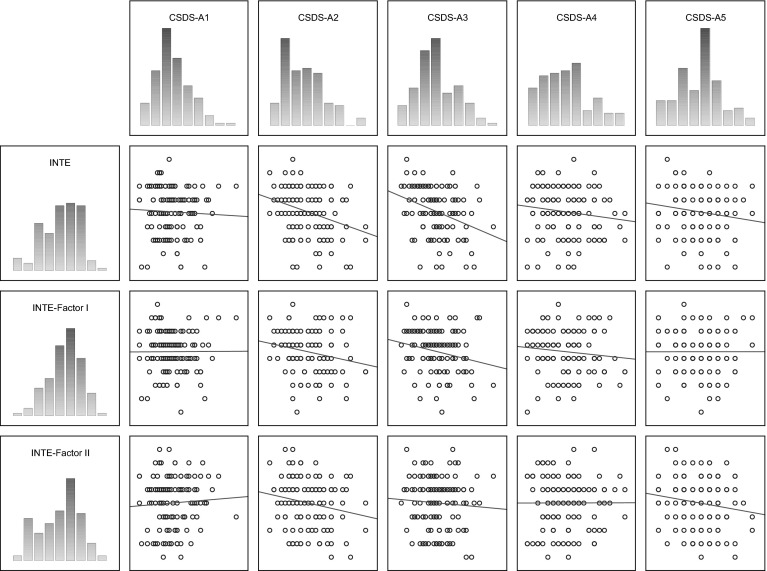
Scatterplot matrix of variables scores (INTE, CS-DS). *CS-DS* Polish version of the “Chronic Self-Destructiveness Scale”,* CS-DS-A1* Transgression and Risk, CS-DS-A2: Poor Health Maintenance,* CS-DS-A3* Social and Personal Neglects,* CS-DS-A4* Lack of Planfulness,* CS-DS-A5* Helplessness and Passiveness in the face of problems/difficulties,* INTE* Polish version of the “Assessing Emotions Scale”,* INTE-Factor I* ability to utilise emotions,* INTE-Factor II* ability to recognise emotions

Table 1 shows correlation coefficients (Kendall’s *τ*) between the studied variables using the CS-DS and INTE; Fig. 1 shows the scatterplot matrices of those scores. Nine statistically significant correlation coefficients were found between results for the variables studied by means of specific CS-DS and INTE scales and indices. Almost all (but one) correlation coefficients, regardless of the significance level, were negative; it was only the correlation coefficient between INTE Factor II and CS-DS scale A1 (Transgression) that was positive, although statistically non-significant.

**Table 1:** Correlation coefficients between variables measured by CS-DS and INTE

Variable	CS-DS-A1	CS-DS-A2	CS-DS-A3	CS-DS-A4	CS-DS-A5
INTE	−0.040 ns	−0.329 p: 0.00000008	−0.325 p: 0.000001	−0.209 p: 0.0003	−0.170 p: 0.001
INTE-Factor I	−0.019 ns	−0.254 p: 00001	−0.216 p: 0.00006	−0.188 p: 0.001	−0.033 ns
INTE-Factor II	+0.096 ns	−0.262 p: 0.0003	−0.035 ns	−0.076 ns	−0.147 0.02

*CS-DS* Polish version of the “Chronic Self-Destructiveness Scale”,* CS-DS-A1* Transgression and Risk, CS-DS-A2: Poor Health Maintenance,* CS-DS-A3* Social and Personal Neglects,* CS-DS-A4* Lack of Planfulness,* CS-DS-A5* Helplessness and Passiveness in the face of problems/difficulties,* INTE* Polish version of the “Assessing Emotions Scale”,* INTE-Factor I* ability to utilise emotions,* INTE-Factor II* ability to recognise emotions

The INTE (total score) correlated significantly (and negatively) with the following four CS-DS scales: A2 (Poor Health Maintenance), A3 (Personal and Social Neglects), A4 (Lack of Planfulness), and A5 (Helplessness and Passiveness in the face of problems/difficulties).

INTE Factor I (ability to utilise emotions in order to support thinking and actions) correlated significantly (and negatively) with the following three CS-DS scales: A2 (Poor Health Maintenance), A3 (Personal and Social Neglects), and A4 (Lack of Planfulness).

INTE Factor II (ability to recognise emotions) correlated significantly (and negatively) with the following two CS-DS scales: A2 (Poor Health Maintenance) and A5 (Helplessness and Passiveness in the face of problems/difficulties).

The highest correlation coefficient occurred between the INTE (general score) and CS-DS A2 (Poor Health Maintenance) being -0.329 (p: 0.00000008). As a matter of fact, it was solely scale CS-DS A2 that correlated with all the INTE variables.

Only scale A1 (Transgression) did not significantly correlate with any INTE variable; while the only positive, although non-significant, correlation coefficient occurred between A1 and INTE Factor II.

The results of these analyses confirm the results of the negative correlations between CS-DS categories and INTE dimensions: the higher the scores for the INTE dimensions, the lower the scores for the CS-DS categories and vice versa: the lower the scores for the INTE, the higher the scores for the CS-DS.

## Discussion

In discussing the results, it will be difficult to refer to results of other research in the area, because no works dealing with the studied issue were found in the available literature.

As already stated, emotional intelligence is a psychological quality (trait, ability) creating favourable conditions for the psychological, social and even physical well-being of the human, whereas the indirect self-destructiveness syndrome is rather harmful to one. Therefore, it can be assumed that emotional intelligence protects against indirect self-destructiveness, while indirect self-destructiveness interferes with, disturbs or even damages both emotional intelligence and human well-being as a whole [8].

In this study, we decided to take a closer look at relationships between specific dimensions or factors of emotional intelligence and specific categories of indirectly self-destructive behaviours.

As already mentioned, all correlations (except for one) carried the minus sign, regardless of the significance level, which can mean that those two types of psychological phenomena (traits, abilities) are in opposition to each other.

In this part of the study, we will deal only with relationships that reached statistical significance.

Poor Health Maintenance (A2) negatively correlated with all dimensions or factors of emotional intelligence. Thus, a conclusion can be drawn that emotional intelligence as a whole, as well as its specific dimensions (components), i.e., ability to recognise emotions and ability to utilise emotions in order to support thinking and actions, protect the individual’s psychophysical health. It is an empirical proof of the fact that there are positive relationships between emotional intelligence and its components and health in general. Individuals with developed emotional intelligence abilities recognise (their own and others’) emotions as well as utilise moods and emotions to motivate adaptive behaviours [4, 8]. That is consistent with the statement that higher emotional intelligence is associated with better psychophysiological health [15, 16]. That may work based on a mechanism, e.g., preventive actions in the case of the so called prodromal asthenia or starting reaction [17] often preceding a medical condition: individuals with higher emotional intelligence can recognise psychological prodromal symptoms of a somatic disease and make attempts at treatment early enough. In turn, in the case of falling ill such a person follows the physician’s instructions and better cooperates with the physician, on the one hand, thanks to the awareness of one’s own state and consequences of one’s own actions but, on the other hand, also owing to the phenomenon of “emotional exchange” with the healthcare professional.

Some psychosocial factors, such as stronger social support and greater satisfaction with social support in individuals with higher emotional intelligence, may serve as buffers against somatic diseases. Moreover, individuals with higher emotional intelligence can, to a larger extent, act according to principles of health behaviour and show better medical compliance [16].

Therefore, individuals with higher emotional intelligence tend to be in a positive mood and easier improve that when they sometimes are in a negative one [15, 16].

Another category of indirectly self-destructive behaviours, Personal and Social Neglects (A3), was also negatively correlated with emotional intelligence in general as well as ability to recognise one’s own emotions and emotions of others. Other authors also report negative correlations between emotional intelligence and deviant social behaviours [18]. Individuals having the most serious problems with respecting others, i.e., prisoners (criminals), have low emotional intelligence [5]. Higher emotional intelligence is associated with better psychosocial functioning, including intrapersonal factors (such as higher optimism) and interpersonal factors (such as better interpersonal, social relations) [16]. Furthermore, individuals with high emotional intelligence show more empathy in relations with others, more self-monitoring in social situation, as well as more closeness and warmth in relations with others [7, 19].

Authors of the emotional intelligence concept report ability to predict, prevent and counteract adjustment disorders such as aggression and violence [20]. A dramatic manifestation of intrapersonal and interpersonal dysfunctions is domestic violence. Perpetrators of domestic violence (mostly males) have lower emotional intelligence than the general population; moreover, emotional intelligence deficits are connected with a tendency to use violence in both the group of violence perpetrators and the general population [21]. Even females suffering domestic violence have lower emotional intelligence than females who do not suffer that [22].

Empathy and self-monitoring in social situations [7, 19] may protect against (prevent) disorders of social and personal functioning. Another well-known researcher of emotional intelligence, Bar-On, also states that emotionally intelligent individuals adapt better in their environment, including the social one [23].

Lack of Planfulness (A4) also negatively correlated with emotional intelligence as a whole and ability to utilise emotions in order to support thinking and actions. Individuals who cannot recognise their own emotions are unable to plan their lives in order to find fulfilment; such planning deficits may lead to feeling the lack of the meaning of life which affects depressive individuals and even those having suicidal ideations [4]. Assuming that academic achievements result also from ability to plan, it was found that emotional intelligence is a good predictor of high achievements at university [5].

Ability to utilise emotions may be helpful in planning one’s own actions and predicting their consequences to be of benefit to oneself and others. That way of acting usually results in better adjustment and more effective coping in the environment [4, 15].

The last category of indirectly self-destructive behaviours, Helplessness and Passiveness in the face of problems/difficulties (A5), negatively correlated with general emotional intelligence and ability to recognise one’s own emotions and emotions of others. Such a result may suggest that emotional intelligence in general, and ability to recognise emotions in particular, protect against the lack of ability to cope with problems and abandoning or refraining from taking remedial measures in difficult situations. The lack of motivation or readiness to take active measures in the face of difficulties or the total abandonment of such measures cause further, secondary, psychological, health-related and social damage. Emotional intelligence protects against depression and the feeling of hopelessness and helplessness [8, 18]. On the other hand, emotional intelligence is connected with greater optimism and the absence of depressive states [7, 8, 19]. The absence of helplessness may be a kind of bridge to psychophysical health. As mentioned earlier, higher optimism and the sense of receiving social support may constitute buffers against a somatic disease [16]. According to Brown and Schutte, emotional intelligence may constitute such protection, too [24]. Some authors [25] propose to call emotional intelligence emotional self-efficacy, while self-efficacy is the opposite or even contradiction of self-handicapping, being one of the major components of indirect self-destructiveness, especially helplessness and passiveness [cf. 8].

It arises from the above deliberations and reasoning that emotional intelligence and its specific components protect against indirectly self-destructive behaviours in the scope of all the studied categories.

As for now, a riddle remains the sole positive (although statistically non-significant) correlation, namely the one between Transgression (CS-DS, A1) and ability to recognise emotions (INTE, Factor II). Perhaps further research will answer the questions why that happens and what that can mean.

## Conclusions

Along with the therapy of existing disorders, the objective of contemporary mental health sciences is more and more often to prevent their occurrence and develop the potential of the individual. An important area within that current can be the scientific identification of psychosocial determinants of human behaviours. Results of the research may prove useful in prophylactic and therapeutic work. The knowledge of relationships between specific categories of indirectly self-destructive behaviours and dimensions of emotional intelligence may allow to orientate psychological measures aimed at preventing behaviours harmful to the human being and improving the quality of his or her life through the strengthening of psychological resources and neutralising risk factors by using emotional intelligence. Improved functioning in the scope of recognising emotions and their utilisation in actions may create favourable conditions for better coping with taking care of one’s own safety, health and development, hence creating favourable conditions for limiting self-destructive behaviours.
